# Supplementary material for: “I just don’t know what will be better other than an apartment which I can’t afford”: Experiences of housing quality following homelessness in Ontario, Canada
Source: PLOS Ment Health. 2025 May 22;2(5):e0000297. doi: 10.1371/journal.pmen.0000297 (PMC12798546; doi:10.1371/journal.pmen.0000297)
Supplement: Attachment — Submitted filename: Summary of Revisions.docx [file pmen.0000297.s002.docx]

| **Summary of Revisions** | |
| --- | --- |
| **Reviewer Feedback** | **Author Response** |
| Reviewer #1: Thank you for asking me to review this well written paper that addresses an important human rights problem in our society. I appreciate the prolonged engagement of the researchers with the population of interest, which can be a hard to reach group. The conclusions are sad but important to expose. | Thank you very much for these sentiments. We agree wholeheartedly, and struggle to see the situation that so many people are experiencing at the moment. Thank you also for your careful attention to our paper, and for the time that you’ve invested in providing us with your feedback. |
| Reviewer #1: The section on recruitment of participants is somewhat unclear. Ethics approval was obtained by the authors of the parent study, but also for the present one? | Thank you for noting this lack of clarity. We have edited the recruitment section of our paper so that it includes the following statement:  “A separate ethics application was not required for the current study, as participants in the parent study provided approval for the conduct of secondary analyses of anonymized transcripts. In the parent study, participants were recruited from organisations providing social service and mental health support to persons with lived experiences of homelessness …” |
| Reviewer #1: Only a subsample of participants from the initial sample was included in the secondary analysis presented in the current paper. Do I understand correctly they were asked for consent for the secondary analyses of the transcripts of the original study? Please clarify this. | Yes. In our informed consent process, participants agreed to allow the principal investigator to provide access to their anonymized transcripts to other researchers for the conduct of such studies: “if in the future, another researcher is interested in using information collected to conduct another research project, we will provide them with the information that we’ve collected, but in a de-identified form.” |
| Reviewer #1: The described analysis matches inductive coding, what do the researchers specifically mean by abductive coding? | Thank you for providing the opportunity for us to describe our processes in further detail. We have added the following statement to the section where we describe our analysis: “We took an interpretivist approach to our analysis and coded interview transcripts abductively, informed by theories of social justice and health equity (25) using thematic analysis (Braun & Clarke, 2014). Abductive coding is a type of coding that explicitly acknowledges a researcher’s theoretical positioning during the process of assigning codes to qualitative data (29). This approach contrasts with inductive coding, which typically involves entering into a dataset with no prior theoretical understandings of the data, or deductive coding, where a theory is used to structure analysis of qualitative data (29).” |
| Reviewer #1: The strategies of trustworthiness mostly pertain to the parent study, the authors should mention this. | We agree that clarifying this for the reader is important. As such, we have contextualized this in the following way as some of the strategies for trustworthiness were only conducted as part of the parent study, while others pertain to the secondary analysis presented in the current paper: “For the parent study, we employed strategies for establishing trustworthiness as identified by Lincoln and Guba (31), which were followed throughout the current study, including:…” |
| Reviewer #1: A few sentences about ethics in the method section might be helpful (in addition to the attachment). For example, did the participants receive anything for their participation and how did the researchers do justice to their efforts? Are results communicated back to participants? | Thank you for this suggestion. We have added additional details to the recruitment section as follows: “All participants were provided with $40 in cash as an honorarium for their involvement in interviews.”  In the data analysis section, we have added the following section:  “Participants who consented to be contacted with the results of the study were provided with a copy of reports and publications resulting from this research using the contact information provided during interviews. Further, organizations that provided services to persons experiencing homelessness in the recruitment cities were provided with all reports and published papers and encouraged to distribute among staff and service users. Further, public presentations were provided in-person and virtually throughout the communities in which we recruited that participants who consented to participate and staff and leadership in organizations that provided services to persons experiencing homelessness were invited to attend.” |
| Reviewer #1: In the results section a table picturing the themes at a glance would be helpful. | Thank you for this helpful suggestion. Instead of a table, we have provided a figure that depicts the four themes and their relationship to the essence of our analysis. See the added attachment (Figure 1). |
| Reviewer #1: In the results section, quotes come with names of participants. Are these their real names? Please clarify (in the text) the choices made in this. | At the end of the recruitment section, we have added this additional detail: “Each participant was asked to provide a pseudonym to protect their confidentiality, which have been used to label quotes in the results section, rather than their real names.”  Thank you for highlighting this omission, which we agree, needs to be included. |
| Reviewer #1: Did researchers in the parent study do a member check to increase validity? If not, this could be mentioned as a limitation. The fact that data were not collected on the basis of the present research question and that analyses were secondary, should also be mentioned. What are possible implications of this? | We did not engage in member checking and agree that this should be listed as a limitation. We also agree that the limitations of a secondary analysis should be mentioned. To respond, we have added this statement to the data analysis section:  “In both the current and the parent study, we did not engage in member checking, as in the experience of the principal investigator, locating participants with experiences of homelessness on more than one occasion can be difficult due the ongoing changes that tend to occur in their lives.”  We have also added this statement to the limitations section at the end of the discussion section:  “While we would have preferred to engage in member-checking with the participants following analysis, we could not do so in the current study, thereby limiting our ability to confirm with participants whether our analysis truly reflected their experiences as they understood them. Further, it is well recognized that secondary analyses are limited in that the research question used for analysis differs from the original research question (38). Readers should be aware that the findings of the current study are limited by the fact that our research question did not focus on housing quality specifically, and important experiences of housing quality following homelessness may not have been identified in our findings. Future research focused specifically on experiences of housing quality following homelessness may provide a more fulsome understanding of this phenomenon to build on the analysis presented in the current study.” |
| Reviewer #2: I would like to begin by congratulating the team on this paper. It addresses an important and relevant problem in your context and highlights the need for more research led by individuals with lived experiences in this area. I believe this work is purposeful and has significant potential for impact. However, there are a few areas that could benefit from further clarification, as outlined below: | Thank you for your kind words, and for providing such a thoughtful review of this work! |
| Reviewer #2:  1. Interviewer Characteristics It would be helpful to provide more information regarding the interviewers’ qualifications. Were all interviewers properly trained, or did they have any prior experience conducting interviews of this nature? | We appreciate you noting the lack of detail in the data collection section. We have added some additional details as follows, which includes information regarding the characteristics of interviewers:  “Participants who met the inclusion criteria of the parent study arranged suitable times with research staff to participate in interviews. Interviews were semi-structured and consisted of questions focused on the conditions needed for individuals to “thrive” following homelessness. Interviewers were research assistants who had received training in the conduct of semi-structured qualitative interviews by the principal investigator and co-investigators of the parent study. Research assistants were persons with lived experiences of homelessness, individuals with experiences of providing service in social service and/or mental health roles with unhoused persons, and/or research students at the master’s or PhD-level who were enrolled in educational programs pertaining to health and rehabilitation fields. Interviews were recorded on a digital recording device and transcribed verbatim. Sample interview questions posed to participants are provided in Table 1.” |
| Reviewer #2:  2. Participant Selection -While the sample size appears optimal, more explicit detail on the sampling method is needed. What type of sampling strategy was employed, and what was the rationale behind it? | Thank you for the opportunity to provide this additional information. We have added the following statement in the recruitment section of our methodology section, which we hope will provide the necessary details (see below, which builds on this):  “We used a purposive sampling strategy in the parent study to generate a sample of individuals who had recent experiences of homelessness. As such, participants were included if they were over the age of 16, and had experienced at least one month of homelessness in the past three years, irrespective of whether they were currently housed or unhoused during interviews.” |
| Reviewer #2:  - There is a noticeable lack of diversity in the sample. The participants are predominantly white, with only two African-American individuals included. Considering that social experiences between racial groups may differ significantly, addressing this limitation or discussing its potential impact on the findings would be beneficial. | We agree that this sample is quite limited, yet reflects the demographic composition of the communities in which we recruited. To account for this, we have added the following statement to the limitation section of the discussion:  “Further, while we tried to recruit participants of a range of ages, races, genders and sexual orientations, we were unable to recruit a sample as diverse as we wished given a lack demographic representation of key groups in the cities in which we recruited. As such, the participants in the current study lacked diversity with respect to race and sexual orientation, and any interpretation of our findings should account for the fact our sample was primarily white and heterosexual. Individuals conducting future studies should seek participation of these underrepresented groups in communities with greater diversity with respect to race and sexual orientation.” |
| Reviewer #2:  -Although I understand that full recruitment details are available in the parent study, i suggest it would be useful to specify how participants were approached for these interviews. Additionally, were there any participants who were approached but declined or were excluded? | We have added the following additional detail to the recruitment section to provide these necessary details:  “We used a purposive sampling strategy in the parent study to generate a sample of individuals who had recent experiences of homelessness. To facilitate recruitment, we arranged suitable times to come to shelters, drop-in centres, encampments, and housing programs with staff in the recruitment organizations. During these times, a research assistant occupied a private interview space where potential participants could ask questions about the study, and where a research assistant could determine eligibility. Further, staff in shelters, drop-in centres, and housing programs were invited to provide the contact of the research team to persons with experiences of homelessness and they were invited to reach out to the team directly via email, telephone or text. None of the participants meeting inclusion criteria were excluded from the study following recruitment.” |
| Reviewer #2:  3. Interview Setting Additional details about the interview process would provide greater clarity. Specifically: -Where and how were the interviews conducted?  -Were all interviews conducted in person? If not, what were the alternative methods (e.g., virtual or phone interviews)?  -On average, how long did the interviews last?  -Could you describe the structure of the interviews and provide more detail about the questionnaire, was a guide used? | We appreciate this suggestion and agree that our previous section on data collection lacked detail. We have responded to this feedback (which was also provided by Reviewer #1 by adding the following to the data collection section of our paper: “Participants who met the inclusion criteria of the parent study arranged suitable times with research staff to participate in interviews. Interviews were semi-structured and consisted of questions focused on the conditions needed for individuals to “thrive” following homelessness. Interviewers were research assistants who had received training in the conduct of semi-structured qualitative interviews by the principal investigator and co-investigators of the parent study. Research assistants were persons with lived experiences of homelessness, individuals with experiences of providing service in social service and/or mental health roles with unhoused persons, and/or research students at the master’s or PhD-level who were enrolled in educational programs pertaining to health and rehabilitation fields. Interviews were recorded on a digital recording device and transcribed verbatim. Sample interview questions posed to participants are provided in Table 1.  TABLE 1 HERE”  Note: The table with the interview questions was included with the original submission.  Further, we have added the following statement at the beginning of the Results section: “Interviews from 19 participants were included in this secondary analysis, and ranged from 11-82 minutes in duration (Mdn=42; IQR=28).”  We hope that this additional satisfies the reviewers’ feedback. Thanks for helping to make our paper stronger, and for us to show the rigor to which we applied to our methods. |
| Reviewer #2:  -It is noted that participants’ names or pseudonyms were used to identify verbatim quotes. While this approach is not common, it would be useful to explain the rationale behind this choice and how it was managed to maintain anonymity and confidentiality. | Thank you for the opportunity to help us to provide clarity around this. Reviewer #1 raised the same consideration. To respond, we have added the following statement to the recruitment section of our paper: “Each participant was asked to provide a pseudonym to protect their confidentiality, which have been used to label quotes in the results section, rather than their real names. A list linking the participant’s pseudonym with their real name was kept by the principal investigator of the parent and the current study, which was kept confidential throughout the research process, by limiting access only to the research team involved in the parent study.” |
| Reviewer #2:  Results: Its unclear which are major and minor themes | We appreciate the request for clarity given that many studies include sub-themes. In our study, we have opted not to include sub-themes, but rather simply an “essence” and “themes” that express the meaning of the essence which is consistent with the method described in Reflexive Thematic Analysis (RTA) (Braun et al.). While sub-themes are consistent with RTA, we did not feel the need to create them to accurately describe the narratives provided by participants. |
