# Supplementary material for: “I just don’t know what will be better other than an apartment which I can’t afford”: Experiences of housing quality following homelessness in Ontario, Canada
Source: PLOS Ment Health. 2025 May 22;2(5):e0000297. doi: 10.1371/journal.pmen.0000297 (PMC12798546; doi:10.1371/journal.pmen.0000297)
Supplement: Attachment — Submitted filename: Summary of Revisions 2.docx [file pmen.0000297.s003.docx]

| **Summary of Revisions** | |
| --- | --- |
| **Reviewer Feedback** | **Author Response** |
| Reviewer #1:  Thank you for your careful consideration of my comments. Congratulations on this important paper. | Thank you for the time you’ve invested in reviewing our paper. We are very grateful for your time and expertise in supporting this work. |
| Reviewer #1:  I have one more minor suggestion; it's about this statement:  “A separate ethics application was not required for the current study, as participants in the parent study provided approval for the conduct of secondary analyses of anonymized transcripts. In the parent study, participants were recruited from organisations providing social service and mental health support to persons with lived experiences of homelessness …”  Since anonymity cannot be guaranteed (someone can always be recognized by the story), I would speak of ‘pseudonymization’. | We appreciate this suggestion and have revised the methods section by changing the word “anonymized” to “pseudonymized” as suggested by Reviewer #1. This segment now reads as:  “A separate ethics application was not required for the current study, as participants in the parent study provided approval for the conduct of secondary analyses of pseudonymized transcripts. In the parent study, participants were recruited from organisations providing social service and mental health support to persons with lived experiences of homelessness …” |
| Reviewer #2: Thank you for your revisions. Authors have addressed all my observations thoroughly and the manuscript is now stronger. | We agree and thank you for your helpful suggestions! We are pleased to hear that we have addressed your previous feedback appropriately. Thank you for the time and expertise invested in this review. We are very appreciative!! |
| Reviewer #2: I suggest some minor revisions be considered in methods section:  -Please consider including that AUDIT and DAST scales were part of the data collection tools. | Thank you for this suggestion. We have revised the methods section to include a discussion regarding the the AUDIT and DAST scales as follows:  “Researchers conducting the parent study also collected demographic information, including details about the mental health of participants. This included the delivery of two standardized measures of substance use including the Alcohol Use Disorder Identification Test-10 (AUDIT-10) (29) and Drug Abuse Screening Test-10 (DAST-10) (30). The AUDIT-10 is a 10-item standardized measure of alcohol use using a 3-5 point nominal scale. The DAST-10 is a 10-item standardized measure of substance use, excluding alcohol, tobacco and caffeine, on a dichotomous (yes/no) scale. Both of these measures have been broadly utilized in health and social care research, and have been demonstrated to be reliable scales with the AUDIT-10 demonstrating internal consistency of 0.75-0.97 in previous research (31) and the DAST-10 demonstrating internal consistency of 0.86-0.90 in previous research (30, 32).”  We hope that the reviewer is satisfied with this inclusion and level of detail. |
| Reviewer #2: -Analysis: Please revise if all citations are in Vancouver format. | We have reviewed our reference list, updated it based on the inclusions above, and have verified that it follows Vancouver style as per the journal specifications. Thanks for this! |
